# Age-Associated DNA Methylation Patterns Are Shared Between the Hippocampus and Peripheral Blood Cells

**DOI:** 10.3389/fgene.2020.00111

**Published:** 2020-03-06

**Authors:** Christopher J. Harris, Brett A. Davis, Jonathan A. Zweig, Kimberly A. Nevonen, Joseph F. Quinn, Lucia Carbone, Nora E. Gray

**Affiliations:** ^1^ Department of Neurology, Oregon Health and Science University, Portland, OR, United States; ^2^ Department of Medicine, KCVI, Oregon Health and Science University, Portland, OR, United States; ^3^ Parkinson’s Disease Research, Education, and Clinical Center, Portland Veteran’s Affairs Medical Center, Portland, OR, United States; ^4^ Department of Molecular and Medical Genetics, Oregon Health and Science University, Portland, OR, United States; ^5^ Department of Medical Informatics and Clinical Epidemiology, Oregon Health and Science University, Portland, OR, United States; ^6^ Division of Genetics, Oregon National Primate Research Center, Beaverton, OR, United States

**Keywords:** aging, DNA methylation, epigenetics, hippocampus, transcription factor

## Abstract

As the population ages, interest in identifying biomarkers of healthy aging and developing antiaging interventions has increased. DNA methylation has emerged as a potentially powerful molecular marker of aging. Methylation changes at specific sites in the human genome that have been identified in peripheral blood have been used as robust estimators of chronological age. Similar age-related DNA methylation signatures are also seen in various tissue types in rodents. However, whether these peripheral alterations in methylation status reflect changes that also occur in the central nervous system remains unknown. This study begins to address this issue by identifying age-related methylation patterns in the hippocampus and blood of young and old mice. Reduced-representation bisulfite sequencing (RBSS) was used to identify differentially methylated regions (DMRs) in the blood and hippocampus of 2- and 20-month-old C57/Bl6 mice. Of the thousands of DMRs identified genome-wide only five were both found in gene promoters and significantly changed in the same direction with age in both tissues. We analyzed the hippocampal expression of these five hypermethylated genes and found that three were expressed at significantly lower levels in aged mice [suppressor of fused homolog (*Sufu*), nitric oxide synthase 1 (*Nos1*) and tripartite motif containing 2 (*Trim2*)]. We also identified several transcription factor binding motifs common to both hippocampus and blood that were enriched in the DMRs. Overall, our findings suggest that some age-related methylation changes that occur in the brain are also evident in the blood and could have significant translational relevance.

## Introduction

As the population of older Americans continues to rise, with estimates that it will more than double in the coming decades (Populations Reference Bureau, 2015), there is a growing interest in understanding the underlying biology of aging in order to identify therapeutic interventions that can help improve the overall health of older people. To this end, there has been an increased focused on epigenetic changes, which are chemical modification of the DNA that do not involve DNA sequence and might affect gene expression. Epigenetic modifications are known to occur throughout the lifespan and can serve as molecular markers of chronological age that can be used to assess and predict age-associated decline and disease, as well as the effects of interventions devised to promote healthy aging (Fahy et al., 2019). DNA methylation in particular has been shown to be a sensitive marker for age, associating so precisely with chronological age that they are often referred to as the “epigenetic clock” (Bollati et al., 2009; Christensen et al., 2009; Maegawa et al., 2010; Rakyan et al., 2010; Alisch et al., 2012; Horvath et al., 2012; Rando and Chang, 2012; Day et al., 2013; Hannum et al., 2013; Horvath, 2013; Johansson et al., 2013; Teschendorff et al., 2013; Weidner et al., 2014).

Evidence of this “epigenetic clock” can be found in almost all tissues and organs, however the brain appears to be the most affected and these changes are thought to contribute to cognitive decline in aging (Fraga et al., 2005; Lardenoije et al., 2015; Starnawska et al., 2017; Barter and Foster, 2018). DNA methylation is known to play an important role in dynamic regulation of gene expression that is involved in synaptic plasticity and learning and memory in aging humans (Borrelli et al., 2008; Day and Sweatt, 2010; Graff and Tsai, 2013; Sweatt, 2013). Rodents similarly display epigenetic alterations as they age (Stubbs et al., 2017; Meer et al., 2018) and many studies have reported that age-related methylation changes in the central nervous system (CNS) can likewise influence synaptic connectivity and cognitive function (Levenson et al., 2006; Miller and Sweatt, 2007; Lubin et al., 2008).

Although methylation changes can be powerful predictors of age-related changes that affect the CNS, evaluating methylation changes in the brain is not clinically feasible. Therefore it is key to identify peripheral DNA methylation patterns that mimic those in the brain region of interest. Similarity of methylation patterns has been reported across many tissue types in both mice and humans (Horvath, 2013; Stubbs et al., 2017). In humans, correlations between age-related methylation changes have been reported for peripheral blood and cerebral cortex (Horvath et al., 2012; Horvath, 2013; Johansson et al., 2013), but not hippocampus, a key region for understanding brain aging and neurodegeneration (Morrison and Hof, 2002; Mattson and Magnus, 2006; Wang and Michaelis, 2010; Moodley and Chan, 2014; Bird, 2017). We consequently focused these studies on the hippocampus, and on methylation changes that were inferred (by location in the promoter) or demonstrated (by concomitant gene expression studies) to be functionally significant.

## Method

### Mice

Six young male C57Bl/6 mice were obtained from Jackson Labs (Bar Harbor, ME) and six aged male C57Bl/6 mice were obtained from the National Institute of Aging Aged Rodent Colony. Mice were maintained in a climate controlled facility with a 12-h light/dark cycle and given food and water ad libitum at the Veteran’s Administration Portland Health Care System (VAPORHCS). At 2 and 20 months of age mice were euthanized *via* CO2 inhalation followed by exsanguination. Blood was collected *via* cardiac puncture and placed in EDTA treated tubes, and hippocampi were dissected and immediately frozen on dry ice. DNA was extracted using DNeasy Blood and Tissue Kit (Qiagen) as per the manufacturer’s instructions. All procedures were conducted in accordance with the NIH Guidelines for the Care and Use of Laboratory Animals and were approved by the institutional Animal Care and Use Committee of the VAPORHCS.

### Reduced-Representation Bisulfite Sequencing Library Generation

RRBS libraries were generated from ~200 ng of genomic DNA extracted from blood and brain. Overnight digestion was performed with the restriction enzyme *MspI* (New England Biolabs). Reaction clean-up was performed with AMPure XP magnetic beads (Beckman Coulter) and library preparation was done with the NEXTflex Bisulfite-Seq Kit (Bioo Scientific Corporation). The digested DNA was end-repaired, A-tailed, and then ligated with the NEBNext Methylated Adaptor (New England Biolabs). Bisulfite conversion was performed with the EZ DNA Methylation-Gold Kit (Zymo Research) before carrying out PCR amplification with NEBNext Multiplex Oligos (New England Biolabs) to barcode each library. A final AMPure XP bead clean was performed and the resulting libraries quantified with the Qubit High Sensitivity dsDNA Assay (Life Technologies, Eugene, OR) and the Bioanalyzer High Sensitivity Analysis (Agilent, Santa Clara, CA). Libraries were multiplexed and sequenced by the OHSU MPSSR on the Illumina NextSeq 500 with the high-output, 75-bp cycle protocol.

### Differential Methylation Analysis

RRBS reads were analyzed for quality with FastQC (v0.11.5), followed by trimming with TrimGalore (v0.5.0) with the “—rrbs” parameter specified. Trimmed reads were aligned to the mouse reference genome from ensemble, version m38 (mm10), with Bismark (v0.19.0) using default parameters. Coverage files output from Bismark were used to obtain CpG methylation rates. The R package methylKit (v1.8.1) was used for differential methylation analysis.

For analysis of differentially methylated regions (DMRs) using methylKit the genome was tiled into 1,000 bp nonoverlapping regions and the CpG methylation rates were averaged over the region. To calculate p-values, a logistic regression model that utilizes a Chi-square test was used to determine if the model with the treatment vector better predicts the outcome variable (methylation proportion) than the null model. P-values were corrected to q-values *via* the SLIM (Wang et al., 2011) method. Overdispersion correction was applied. The dataset was reorganized into blood samples and hippocampus samples in order to compare old to young within each tissue.

All DMRs with a q-value < 0.05 and a methylation difference > 10% were compiled and overlapping genes were annotated using a custom pipeline. DMRs were considered as occurring within a promoter region, intron, or exon based on using ensemble annotation for mouse (specifically, GRCm38.92). We defined promoters as 3 kb upstream from a transcription start site (TSS). If the DMR overlapped an annotated promoter, exon, or intron, then it was considered as overlapping that given gene feature. In some instances a DMR can overlap more than one feature; for example, a DMR that spanned the promoter region and the first exon of a gene would be considered as overlapping both features. For intergenic DMRs, the closest gene and the distance between the DMR and TSS were also annotated.

### Characterization of DMRs

In order to identify pathways enriched in DMRs, we used the publicly available tools GREAT (http://great.stanford.edu/public/html/) and GOrilla (http://cbl-gorilla.cs.technion.ac.il/). To identify pathways enriched in DMRs occurring specifically in the promoter region we used the PANTHER (http://www.pantherdb.org/). These can be found in **Supplemental Tables 1** and **2**.

We then compared DMRs occurring in the blood and hippocampus identifying those in the same location that were changed in the same direction in both tissue types. We also performed a permutation analysis to determine if those common DMRs we identified were merely due to random chance. Randomly selected 1,000 bp regions were used in differential analysis, choosing 1,528 regions for blood and 740 regions for hippocampus out of the ~96,000 used in the initial differential analysis. We then assigned the randomly selected regions a fold change direction, using the same number of hypomethylated and hypermethylated regions as in the original analysis. Finally, we determined how many regions were shared while also possessing the same fold change directions between the two shuffled datasets. This process was repeated 1,000 times, and the distribution of the number of regions shared and in the same direction was determined.

Finally, motif enrichment was performed on the significant DMRs from each tissue type using HOMER (Hypergeometric Optimization of Motif EnRichment) specifying the use of the given size of the regions and normalizing for CpG content against the random background. Enriched motifs with a Benjamini value of less than 0.05 that were common between the blood and the hippocampus were identified.

### Gene Expression Analysis

The contralateral hippocampus not used for DNA extraction was used for RNA-extraction and qPCR. RNA was extracted using Tri-reagent (Molecular Research Center) and reverse transcribed using Superscript III First Strand Synthesis kit (Thermo Fisher Scientific) as per the manufacturers’ instructions. Relative gene expression was determined using TaqMan Gene Expression Master Mix (Invitrogen) and commercially available TaqMan primers (Invitrogen) for suppressor of fused homolog (Sufu), potassium voltage-gated channel subfamily E member 1 (Kcne1), nitric oxide synthase 1 (Nos1), tripartite motif containing 2 (Trim2), gamma-glutamyltransferase 1 (Ggt1), and glyceraldehyde-3phosphate dehydrogenase (GAPDH). Quantitative PCR (qPCR) was performed on a StepOne Plus Machine (Applied Biosystems) and analyzed using the delta-delta Ct method. Statistics were analyzed using GraphPad software using a two-tailed t-test.

## Results

### Differentially Methylated Regions Were Detectable in Both the Blood and Hippocampus of Aged Mice

**Figure 1:**
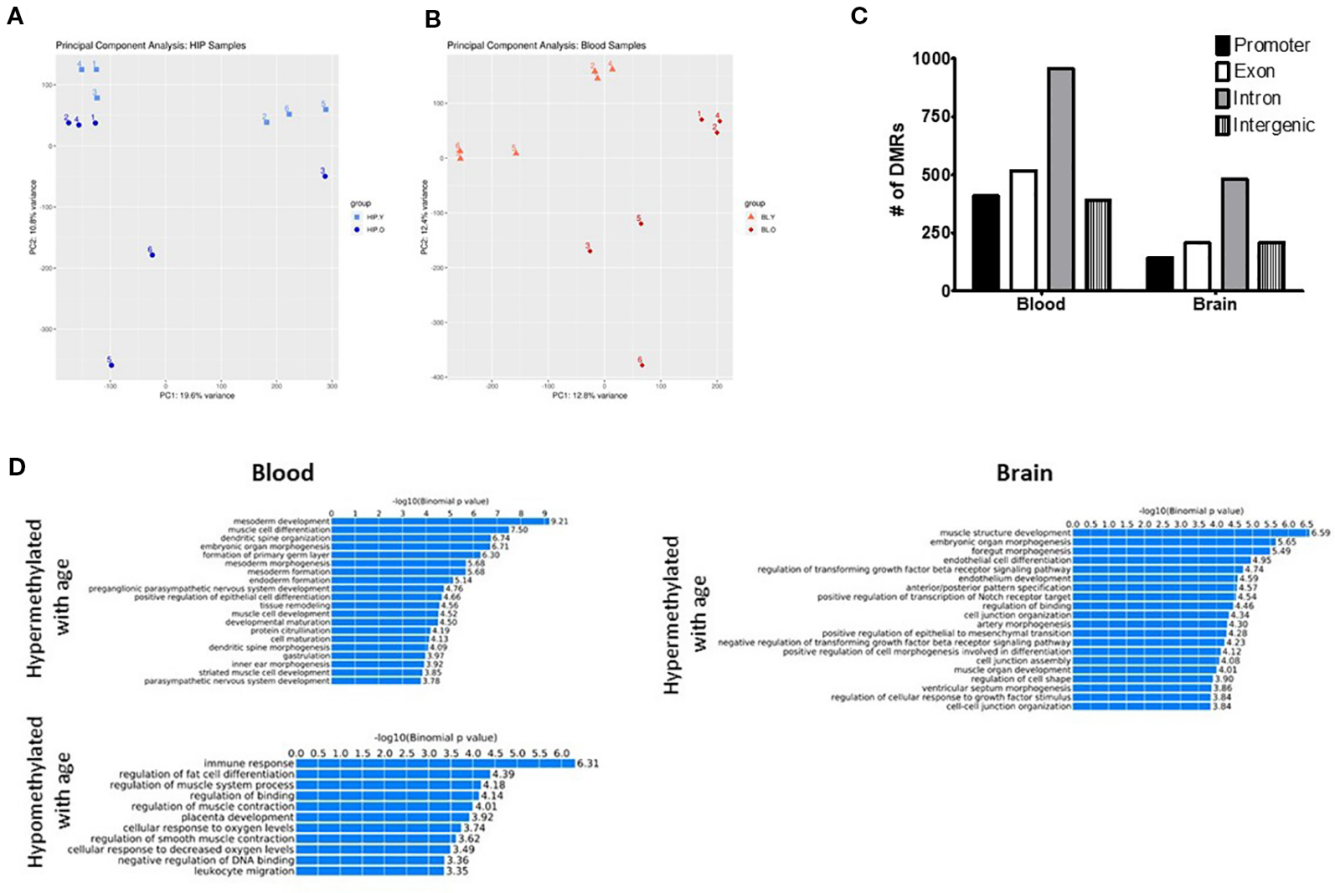
Principal component analysis (PCA) of CpG methylation genome-wide showed separation between young and old mice for both the hippocampus **(A)** and blood **(B)**. **(C)** Differentially methylated regions (DMRs) were identified in promoter, intron, exon, and intragenic locations in both the blood and hippocampus. **(D)** GO analysis identified several biological processes associated with both hyper- and hypomethylated DMRs in each tissue.

In order to identify possible differences in DNA methylation between young and old mice in hippocampus and blood we generated RRBS data for both young (2 months) and aged (20 month) mice and both tissues. The choice of RRBS was based on the possibility to obtain genome wide data at a fraction of the cost than whole genome bisulfite sequencing. This method allows for the capture CpG methylation in promoters, CpG islands, and gene bodies. Using all CpGs present with at least 10X coverage in all datasets we performed Principal Component Analysis (PCA) to assess global differences between our groups (see **Supplementary Table 1** for CpG numbers at different coverages). The PCA plot showed good separation between young and old mice in both the hippocampus (**Figure 1A**) and the blood (**Figure 1B**), suggesting that genome-wide differences in methylation occur with aging, independently from the tissue. Because samples were analyzed on two separate sequencing runs (with tissue type and age randomized) some clustering did occur within each run. Therefore, sequencing run was also included as a covariate during differential analysis.

Next, we identified differentially methylated regions (DMRs) between old and young animals, including differences in methylation >10% (q-value < 0.05) and identified 740 DMRs in the hippocampus and 1,528 in the blood. The majority of DMRs in each tissue were found in intronic regions; however, DMRs were identified in promoters and exons as well as intergenic regions (**Figure 1C**). In order to determine if DMRs were enriched in specific pathways, we used the GREAT toolkit (McLean et al., 2010). Specifically, to carry out this analysis we isolated the DMRs promoters and separated them into hyper- or hypomethylated with age (**Figure 1D**). While there were no biological processes associated with hypomethylated DMRs in the brain, we identified enrichment in pathways involved in immune response and cell differentiation in the blood. Hypermethylated DMRs were enriched in mesoderm development and muscle cell differentiation processes in blood and muscle development and organ morphogenic processes in the hippocampus. We additionally used PANTHER (Thomas et al., 2003) as a different approach to find pathways that were associated with genes containing DMRs in the promoter region that were either hypo- or hypermethylated with age in each tissue (**Supplementary Tables 2** and **3**).

### Common DMRs Between Hippocampus and Blood of Aged Mice Display Changes in Gene Expression

**Figure 2:**
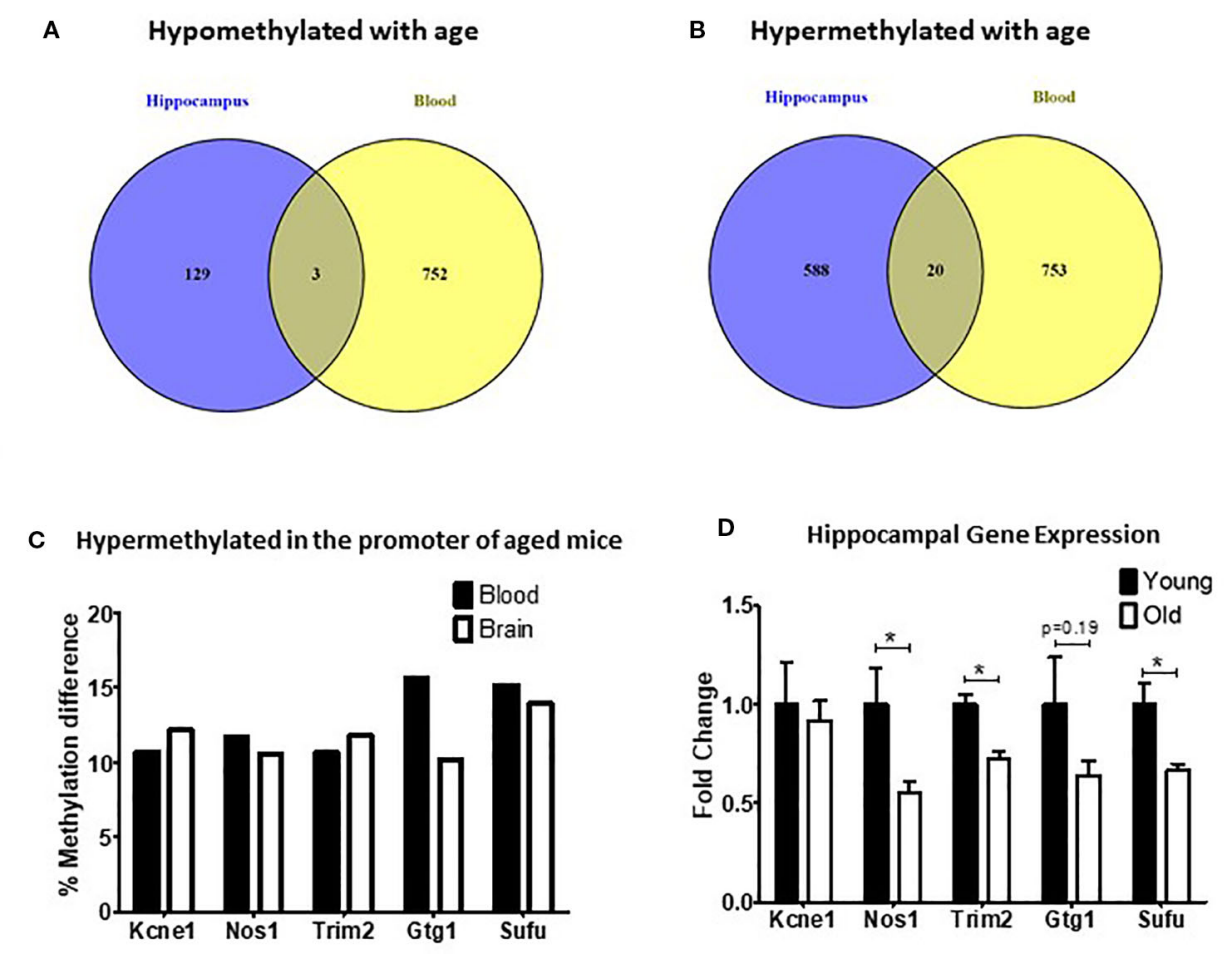
Differentially methylated regions (DMRs) shared between blood and hippocampus that were either hypomethylated **(A)** or hypermethylated **(B)** in aged mice. **(C)** The percent methylation difference between the five common genes that were hypermethylated in the same location in the promotor was similar in both the blood and the brain. **(D)** Three out of five genes with common hypermethylated DMRs in the promoter showed reduced expression in the hippocampus (*p < 0.05, n = 6).

DNA methylation is one of the main mechanisms that regulate gene expression. In order to determine if changes in DNA methylation were also associated with changes in gene expression, we first identified the DMRs shared between hippocampus and blood. Although there was a high correlation between methylation changes in the blood and hippocampus (**Supplementary Figure 1**), we found only three DMRs shared across tissues that were both hypomethylated with age (**Figure 2A**) and 20 that were hypermethylated with age (**Figure 2B**). Through a permutation analysis we determined that that the number of shared DMRs identified here (N = 23) is significantly greater than the number of shared regions that would be obtained by random chance (**Supplementary Figure 2**).

Of the DMRs shared between the blood and hippocampus that changed in the same direction we decided to focus on the five DMRs that were located in promoters because of the strong association between promoter methylation changes and alterations in gene expression (Jones, 2012). In all five cases promoters were found to be hypermethylated with age (**Figure 2C**). We found that the expression of three of the five genes (*Nos1*, *Trim2*, and *Sufu*) was significantly repressed in the hippocampus of aged mice with a fourth (*Gtg1*) showing a nonsignificant trend toward repression as well. No change was observed in the hippocampal expression of *Kcne1* between old and young animals (**Figure 2D**).

### Several Transcription Factor Binding Sites Were Enriched in DMRs in Both the Blood and the Brain of Aged Mice

DMRs can correspond to regulatory regions in the genome where changes in methylation might vary affinity for binding of transcription factors (TF). In order to investigate the possibility that age-related differential methylation might impact binding of biologically relevant TF, we employed the Hypergeometric Optimization of Motif EnRichment (HOMER) tool (Heinz et al., 2010; Populations Reference Bureau, 2015). We identified 54 TF binding motifs enriched in DMRs in the hippocampus and 111 in blood and 14 of these were common to both tissues (**Table 1**).

**Table 1:** Transcription factor binding sites significantly enriched in differentially methylated regions (DMRs) and shared by blood and the brain.

Name	q-value (Benjamini)	% of Target Sequences with Motif	q-value (Benjamini)	% of Target Sequences with Motif
Elk4	<0.0000	36.4	<0.0000	35.6
Elk1	<0.0000	33.1	<0.0000	34.1
Elf1	<0.0000	30.1	<0.0000	30.2
Fli1	<0.0000	53.8	<0.0000	52.8
ETV4	<0.0000	55.8	<0.0000	54.4
Zac1	0.0005	86.0	0.0011	84.5
GABPA	0.0032	44.9	<0.0000	45.6
ETA	0.0039	19.1	<0.0000	19.4
AT5G05550	0.0065	51.8	0.0001	53.2
ETV1	0.0176	62.2	0.0012	61.2
AT3G58630	0.0300	6.0	0.0137	6.2
RXR	0.0261	53.8	0.0197	51.4
EAR2	0.0261	58.2	0.0001	57.13
RAP211	0.0357	66.1	0.0003	68.6

## Discussion

In this study, we evaluated the hippocampus and peripheral blood of 2- and 20-month-old C57Bl/6 male mice to try and identify commonalities in the methylation pattern between the two tissues that occur with aging. CpG methylation between the blood and the hippocampus was found to be highly correlated (**Supplementary Figure 1**). This is consistent with previous reports of correlations between the blood and other brain regions in humans (Horvath et al., 2012). We found thousands of significant DMRs in each tissue distributed in promoters, gene bodies, and intergenic locations. Pathway analysis of these DMRs revealed that in the blood hypomethylated DMRs were enriched in immune response and regulation of fat cell differentiation processes while hypermethylated DMRs were enriched in mesoderm development and muscle cell differentiation processes. In the brain, there were no biological processes identified to be enriched among the hypomethylated DMRs while the hypermethylated DMRs were enriched in muscle structure development and embryonic organ morphogenic processes.

Among these DMRs, relatively few were shared and changed in the same direction in both the hippocampus and blood (3 hypomethylated and 20 hypermethylated) and only five were located in the promoter region, all of which were hypermethylated (**Figure 2**). Hypermethylation of promoters is usually associated with repressed transcription and reduced gene expression (Jones, 2012). We consequently decided to focus our gene expression analysis on the five genes with hypermethylated DMRs in the promoter region: *Kcne1*, *Nos1*, *Trim2*, *Gtg1*, and *Sufu*. Although *Kcne1* expression was unchanged, hippocampal expression of *Nos1*, *Trim2,* and *Sufu* was all significantly reduced in the aged animals and *Gtg1* showed a similar, but nonsignificant, trend as well.

In addition to being a marker for brain aging that can be detected peripherally, these gene expression changes could also have functional relevance. Of note, expression changes of two of the three genes found to be significantly reduced in expression with age, *Nos1* and *Trim2*, have also been reported to be associated with changes in synaptic plasticity and cognitive function. Mice lacking *Nos1* gene have been shown deficits in executive function and spatial memory (Weitzdoerfer et al., 2004; Zoubovsky et al., 2011) and in humans, *NOS1* mutations have been identified as risk factor for schizophrenia, particularly with schizophrenia with profound impairment in cognitive function (Zhang et al., 2015).

*Trim2* expression has also been associated with neuronal health. *Trim2* is an E3 ubiquitin ligase that is highly expressed in the hippocampus and deficiencies in Trim2 levels has been associated with axonal neuropathy (Ylikallio et al., 2013). Additionally neurofilament-light (NF-L) protein is a target of Trim2 that is involved in dendritic branching and dendrite spine formation and it has been reported that mutations or loss of *Trim2* leads to accumulation of NF-L resulting in progressive neurodegeneration and synaptic loss (Ylikallio et al., 2013). NF-L levels have been reported to be elevated in the cerebrospinal fluid (CSF) and blood of patients with synucleinopathies, tauopathies, and Alzheimer’s disease and similar increases can been seen in the CSF and plasma of mouse models of the same diseases (Bacioglu et al., 2016).

By performing a motifs search on all DMRs, we identified 14 TF motifs (**Table 1**) that were significantly enriched in age-related DMRs in both the blood and brain suggesting potential translational relevance of the findings from this study. Six of those 14 common motifs (ELK1, ELF1, ETV1, ETV4, ETV5, RXR) have been associated with modulating synaptic density, arborization, and neurite outgrowth (Gao et al., 1996; Abe et al., 2011; Szatmari et al., 2013; Boone et al., 2017). Furthermore, ETV1, ELK1, EAR2, RXR, and GABPA have been implicated in regulating cognitive function. Specifically, ELK1 has been reported to be involved in transcriptional regulation of several key immediate early genes required for synaptic plasticity and subsequent memory formation (Minatohara et al., 2015; Hullinger and Puglielli, 2017) while loss of EAR2 has been shown to cause learning and memory deficits in healthy mice and exacerbates those seen in mouse models of Alzheimer’s disease (Morrison and Hof, 2002). Changes in ETV1 expression have likewise been linked with cognitive decline (Abe et al., 2011; Ding et al., 2016). RXR agonists that increase binding to the RXR motif have been shown to enhance synaptic density and improve cognitive function in mouse models of aging and Alzheimer’s disease (Cramer et al., 2012; Nam et al., 2016; Tachibana et al., 2016; Mariani et al., 2017). The binding partner of GABPA, METTL23, has been reported to play an essential role in human cognition (Reiff et al., 2014). Additionally, the GABPA binding was also recently found to be enriched in the frontal cortex of aged humans and was determined to be a potential regulator of aging-related genes (Meng et al., 2016). As DNA methylation is able to change binding affinity of TF to enhancers and regulatory sequences, our findings suggest that age-related changes in methylation might affect gene networks whose regulation depends on some of these TFs. More functional studies will be needed to validate this hypothesis.

In conclusion, we were able to identify a methylation signature in the hippocampus of aged mice that was also present in the blood. Interestingly, some of these age-related differences occur in genes relevant to neuronal health, synaptic density and cognitive function. Additional experiments are needed to confirm these results are also relevant in aged female mice, qs some epigenetic modifications might be sex specific (Masser et al., 2017; Gegenhuber and Tollkuhn, 2019). If a common methylation footprint is also be found between the blood and the hippocampus of female animals, it would be of interest in future work to determine if antiaging or cognitive enhancing interventions can modify these changes in both sexes. Ultimately confirming these methylation changes in a human population will be necessary if this is to be adapted for clinical use.

## Data Availability Statement

The datasets generated for this study are available on request to the corresponding author.

## Ethics Statement

The animal study was reviewed and approved by institutional Animal Care and Use Committee of the VAPORHCS.

## Author Contributions

NG, LC, and JQ contributed to the conception and design of the study. JZ and KN isolated the DNA and prepared the sequencing libraries. BD performed the data analysis. CH performed the hippocampal gene expression analysis. NG and CH wrote the first draft of the manuscript and BD and LC also wrote sections of the manuscript. All authors approved the submitted version.

## Funding

This work was funded by NIH-NCCIH grant R00AT008831 to NG, an OHSU Faculty Innovation grant awarded to NG, and a Department of Veterans Affairs Merit Review grant awarded to JQ. LC is supported by R01HG010333 and the NIH/OD P51 OD011092 to the Oregon National Primate Research Center.

## Conflict of Interest

The authors declare that the research was conducted in the absence of any commercial or financial relationships that could be construed as a potential conflict of interest.
