# Supplementary figures and images for: Age-Associated DNA Methylation Patterns Are Shared Between the Hippocampus and Peripheral Blood Cells
Source: Front Genet. 2020 Mar 6;11:111. doi: 10.3389/fgene.2020.00111 (PMC7067920; doi:10.3389/fgene.2020.00111)

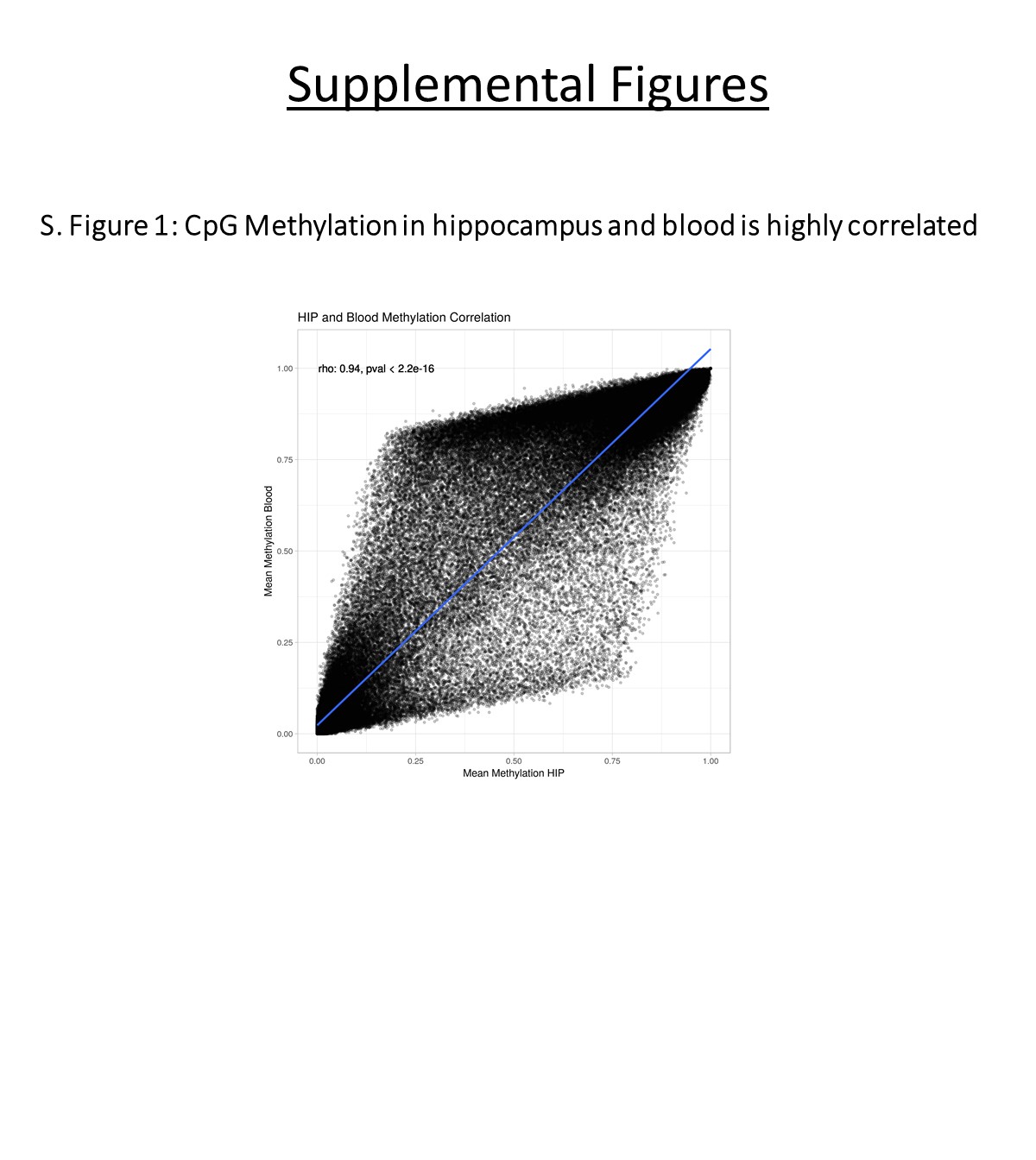

Supplement: Supplementary file 1 [file Image_1.jpg]

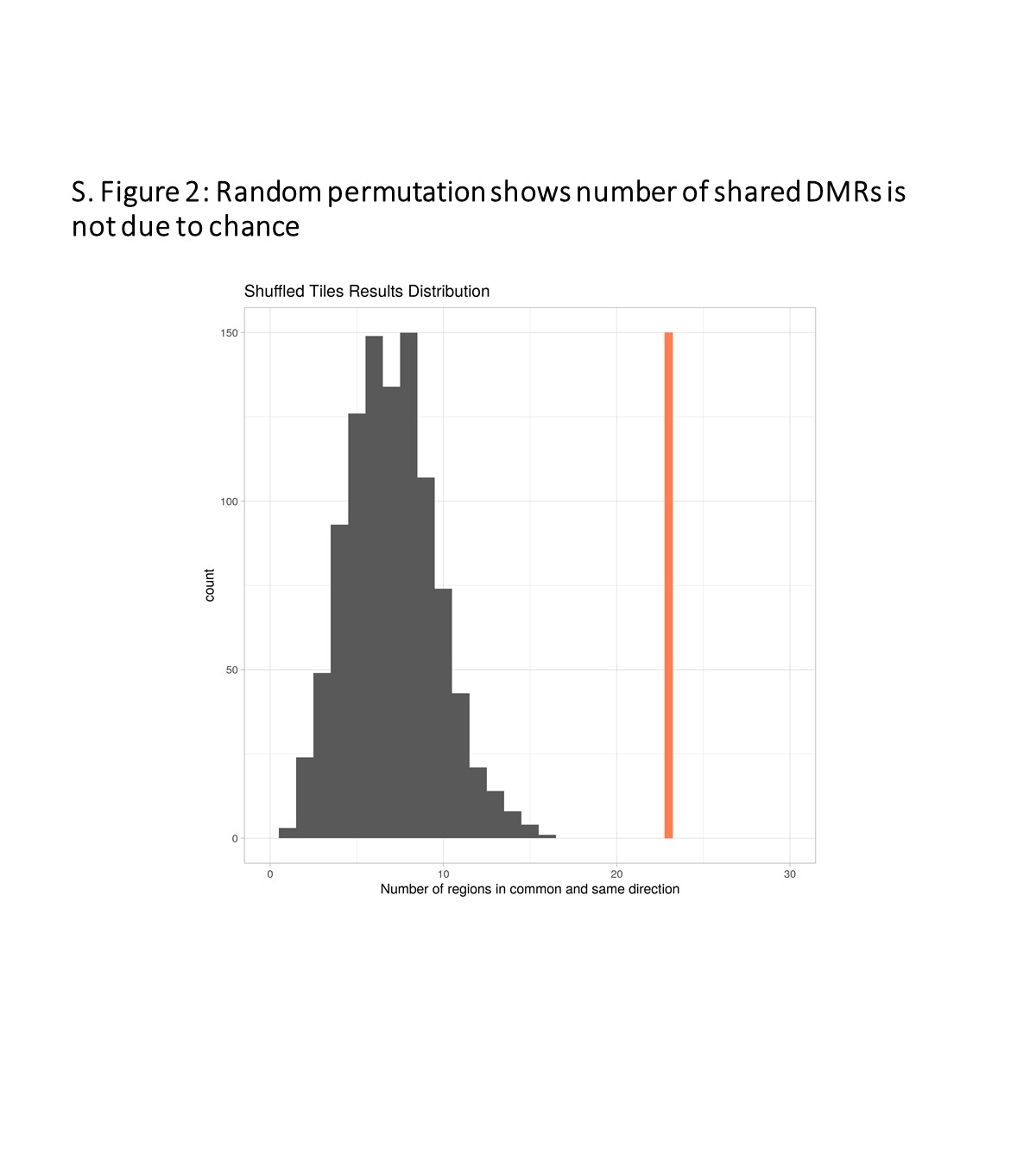

Supplement: Supplementary file 2 [file Image_2.jpg]
