# Supplementary material for: Age-Associated DNA Methylation Patterns Are Shared Between the Hippocampus and Peripheral Blood Cells
Source: Front Genet. 2020 Mar 6;11:111. doi: 10.3389/fgene.2020.00111 (PMC7067920; doi:10.3389/fgene.2020.00111)
Supplement: Supplementary file 3 [file Table_1.docx]

S. Table 1. Number of CpGs at Various Coverages

| Sample name | 1x | 10x | 40x |
| --- | --- | --- | --- |
| Young hippocampus 1 | 2,226,042 | 557,214 | 222,563 |
| Young hippocampus 2 | 1,827,233 | 575,262 | 250,321 |
| Young hippocampus 3 | 2,015,774 | 624,465 | 279,234 |
| Young hippocampus 4 | 1,920,473 | 587,741 | 261,080 |
| Young hippocampus 5 | 2,132,290 | 565,065 | 238,176 |
| Young hippocampus 6 | 1,916,212 | 615,425 | 257,896 |
| Old hippocampus 1 | 1,923,552 | 601,047 | 267,445 |
| Old hippocampus 2 | 1,923,552 | 601,047 | 267,445 |
| Old hippocampus 3 | 1,934,103 | 565,636 | 240,091 |
| Old hippocampus 4 | 2,159,719 | 620,883 | 293,691 |
| Old hippocampus 5 | 1,713,984 | 500,564 | 200,631 |
| Old hippocampus 6 | 1,625,074 | 538,312 | 206,357 |
| Young blood 1 | 2,366,572 | 611,539 | 278,673 |
| Young blood 2 | 2,256,019 | 612,075 | 24,252 |
| Young blood 3 | 2,166,138 | 546,918 | 224,498 |
| Young blood 4 | 2,351,130 | 592,174 | 253,693 |
| Young blood 5 | 2,208,873 | 561,824 | 237,469 |
| Young blood 6 | 1,909,881 | 509,634, | 198,891 |
| Old blood 1 | 2,223,314 | 605,673 | 276,735 |
| Old blood 2 | 2,471,773 | 611,753 | 271,503 |
| Old blood 3 | 2,003,889 | 538,273 | 221,427 |
| Old blood 4 | 2,319,508 | 586,294 | 254,898 |
| Old blood 5 | 1,964,097 | 52,757 | 215,646 |
| Old blood 6 | 2,121,687 | 562,778 | 234,570 |
