# Supplementary material for: Age-Associated DNA Methylation Patterns Are Shared Between the Hippocampus and Peripheral Blood Cells
Source: Front Genet. 2020 Mar 6;11:111. doi: 10.3389/fgene.2020.00111 (PMC7067920; doi:10.3389/fgene.2020.00111)
Supplement: Supplementary file 4 [file Table_2.docx]

S. Table 2: Pathway blood promotors PANTHER

| **Hypomethylated** | |
| --- | --- |
| P53 pathway | Sumo3 |
| Cadherin signaling pathway | Ptpn1 |
| Angiogenesis | Shc1 |
| Glycolysis | Aldoc |
| Integrin Signalling pathway | Shc1, Itga2 |
| Inflammation Mediated by chemokine and cytokine signaling pathway | Itga2,Shc1, Grk2 |
| Synaptic vesicle trafficking | Stx1a |
| EGF receptor signlaing pathway | Shc1 |
| Parkinson's disease | Grk2 |
| Beta1 adrenergic receptor signaling pathway | Ryr3 |
| Heterotrimeric G-protein signaling pathway-rod outer segment phototransduction | Cngb1 |
| Ras pathway | Shc1 |
| FAS signaling pathway | Gsn |
| Metabotropic glutamate receptor group III pathway | Stx1a |
| Axon guidance mediated by netrin | Unc5b, Unc5a |
| Alzheimer's diesase - presenilin pathway | Bace1, Pcsk6 |
| Nicotinic acetylcholine receptor signaling pathway | Stx1a |
| Heterotrimeric G-protein signaling pathway-Gi alpha and Gs alpha mediated pathway | Grk2 |

| **Hypermethylated** | | |  |  |
| --- | --- | --- | --- | --- |
| Axon guidance mediated by Slit/Robo | Ntng2 | |  |  |
| Angiogensis | Wnt10b, Angpt2, Pdgfra | |  |  |
| Dopamine receptor mediated signaling pathway | Ppp1r1b | |  |  |
| Nicotine pharmacodynamics pathway | Ppp1r1b | |  |  |
| O-antigen biosynthesis | Gfpt2 | |  |  |
| Hedgehog signaling pathway | Sufu | |  |  |
| Endothelin signaling pathway | Adcy3 | |  |  |
| Integrin signaling pathway | Col16a1, Itga3, Col9a3, Lims2 | |  |  |
| Inflammation mediated by chemokine and cytokine signaling pathway | Grap2, Nfatc4 | |  |  |
| Metabotropic glutamate receptor group I pathway | Grin3a | |  |  |
| Wnt signaling pathway | Cdh7, Wnt10b, Cdh8, Naftc4 | |  |  |
| EGF receptor signaling pathway | Spry4 | |  |  |
| Metabotropic glutamate receptor group III pathway | Grin3a | |  |  |
| Axon guidance mediated by netrin | Ntng2, Nfatc4 | |  |  |
| B Cell activation | Nfatc4 | |  |  |
| Fructose metabolism | | | Aldoc | |
| CCKR signaling map | | | Shc1, Ryr3 | |
| Beta2 adrenergic receptor signaling pathway | | | Ryr3 | |
| Huntington disease | | | Capn5 | |
| Metabotropic glutamate receptor groupII pathway | | | Stx1a | |
| AngiotensisII-stimulated signaling through G proteins and beta-arresting | | | Grk2 | |
| Alzheimer's disease - amyloid secretase pathway | | | Bace1, Pcsk6 | |
| Interleukin signaling pathway | | | Shc1 | |
| FGF signaling pathway | | | Shc1 | |
| Heterotrimeric G-protein signaling pathway -Gqalpha and Go alpha mediated pathway | | | Grk2, Rap1gap | |
| Muscarinic acetylcholine receptor 1 and 3 siganling pathway | | | Stx1a | |
| Gonadotropin releasing hormone receptor pathway | | | Gata2 | |
| TGF-beta signaling pathway | | | Lefty1 | |
| Muscarinic acetylcholine receptor 2 and 4 siganling pathway | | | Stx1a | |
| PDGF signaling pathway | | | Shc1 | |

| Alzheimer's disease - presenilin pathway | Wnt10b, Trim2 |
| --- | --- |
| Notch signaling pathway | Hes5 |
| Nicotinic acetylcholine receptor signaling pathway | Myo18a |
| Heterotrimeric G-protein signaling pathway - Gialpha and Gs alpha mediated pathway | Gpsm2, Adcy3 |
| CCKR signaling map | Nos1 |
| Hungtington's disease | Tubb4a, Sin3b, Dctn1, Grin3a, Dlg4 |
| T cell activation | Grap2, Zap70, Nfatc4 |
| Ionotropic glutamate receptor pathway | Grin3a |
| N-acetylglucosamine metabolism | Gfpt2 |
| FGF signaling pathway | Spry4, Fgf17 |
| Gondaotropin releasing hormone receptor pathway | Otx1, Nos1, Nfatc4, Adcyap1r1 |
| Muscarinic acetylcholine receptor 1 and 3 signaling pathway | Grin3a |
| Heterotrimeric G-protein signaling pathway - Gqalpha and Go alpha mediated pathway | Gpsm2 |
| TGF-beta signaling pathway | Gdf2 |
| blood coagulation | Gp1bb |

| PDGF signaling pathway | Pdgfra. Grap2 |
| --- | --- |
| Cytoskeletal regulation by Rho GTPase | Tubb4a, Pfn3 |
| Cadherin signaling pathway | Cdh8, Wnt10b, Cdh7 |
