# Supplementary material for: Age-Associated DNA Methylation Patterns Are Shared Between the Hippocampus and Peripheral Blood Cells
Source: Front Genet. 2020 Mar 6;11:111. doi: 10.3389/fgene.2020.00111 (PMC7067920; doi:10.3389/fgene.2020.00111)
Supplement: Supplementary file 5 [file Table_3.docx]

S. Table 3: Pathways Brain Promoters PANTHER

| **Hypomethylated** | |
| --- | --- |
| Cytoskeletal regulation by Rho GTPase | Vasp |
| Angiogeneiss | Cryab, Pld2 |
| Parkinson's disease | Pld2 |
| p38 MAPK | Mef2d |
| Gonadotropin releasing hormone receptor pathway | Nr4a1 |
| Ras pathway | Pld2 |
| CCKR signaling map | Nr4a1, Mef2d |
| Integrin signaling pathway | Vasp |
| Oxidative stress response | Dusp26 |
| Axon guidance mediated by netrin | Vasp |
| VEGF signaling pathway | Cryab |
|  |  |
|  |  |
|  |  |
|  |  |
|  |  |

| **Hypermethylated** | |
| --- | --- |
| Valine biosynthesis | Bcat1 |
| Cadherin signaling pathway | Pcdh8 |
| Nicontinic acetylcholine receptor signaling pathway | Gripap1 |
| Heterotrieric G-protein signaling pathway - Gialpha and Gs alpha mediated pathway | Adora2a |
| Hedgehog singaling pathway | Sufu |
| Endothelin signaling pathway | Edn2 |
| CCKR signaling map | Nos1 |
| Adenine and hypoxanthine salvage pathway | Aprt |
| Wnt signaling pathway | Pcdh8 |
| Heterotrieric G-protein signaling pathway - Gq alpha and Go alpha mediated pathway | Adora2a |
| Gonadotropin releasing hormone receptor pathway | Nos1, Smad3, Dgkz |
| TGF-beta signaling pathway | Smad3 |
| Leucine biosynthesis | Bcat1 |
| Alanaine biosynthesis | Bcat1 |
| Isoleucine biosynthesis | Bcat1 |
| Alzheimer's disease - presinilin pathway | Trim2 |
